# Bilateral facial nerve palsy associated with amphiphysin antibody in metastatic breast cancer: a case report

**DOI:** 10.1186/s13256-021-02727-3

**Published:** 2021-03-26

**Authors:** Vineet Kwatra, Michail Charakidis, Narayan V. Karanth

**Affiliations:** Medical Oncology Department, Alan Walker Cancer Centre, Royal Darwin Hospital, Northern Territory, Australia

**Keywords:** Facial nerve palsy, Bell’s palsy, Amphiphysin antibody, Breast cancer, Nab-paclitaxel, Paraneoplastic syndrome

## Abstract

**Background:**

Paraneoplastic neurological syndrome is an immune-mediated phenomenon where antibodies from tumor cells are produced against neuronal proteins. Amphiphysin antibody is an onconeural antibody linked to the diagnosis of breast cancer and small-cell lung cancer. It is uncommon and typically associated with stiff-person syndrome, of which 90% of patients are eventually diagnosed with breast cancer.

**Case presentation:**

We present a case of a 47-year-old Caucasian woman with metastatic hormone receptor-positive breast cancer who developed bilateral facial nerve palsy while on treatment with nab-paclitaxel. The patient was found to have anti-amphiphysin antibody in the serum and cerebrospinal fluid. She was treated with methylprednisolone and intravenous immunoglobulin, which resulted in partial improvement in her facial nerve palsy.

**Conclusions:**

This case highlights a rare presentation of bilateral facial nerve palsy that likely related to paraneoplastic syndrome associated with the presence of anti-amphiphysin antibody.

## Background

Paraneoplastic neurological syndrome (PNS) is an immune-mediated phenomenon in which antibodies respond against neuronal proteins produced by tumor cells (onconeural antibodies) [1]. The presence of onconeural antibodies is a useful diagnostic marker of PNS [2]. They are specific to a group of malignant diseases rather than identified as a neurological syndrome [3]. An amphiphysin antibody is an onconeural antibody that has been identified and linked to the diagnosis of breast cancer and small-cell lung cancer (SCLC) [4–6].

We describe the first case in the literature of bilateral facial nerve palsy with the presence of anti-amphiphysin antibodies in a patient diagnosed with metastatic hormone receptor-positive, estrogen receptor (ER)/progesterone receptor (PR) positive, human epidermal growth factor receptor 2 (HER2)-negative breast cancer.

## Case presentation

**Fig. 1:**
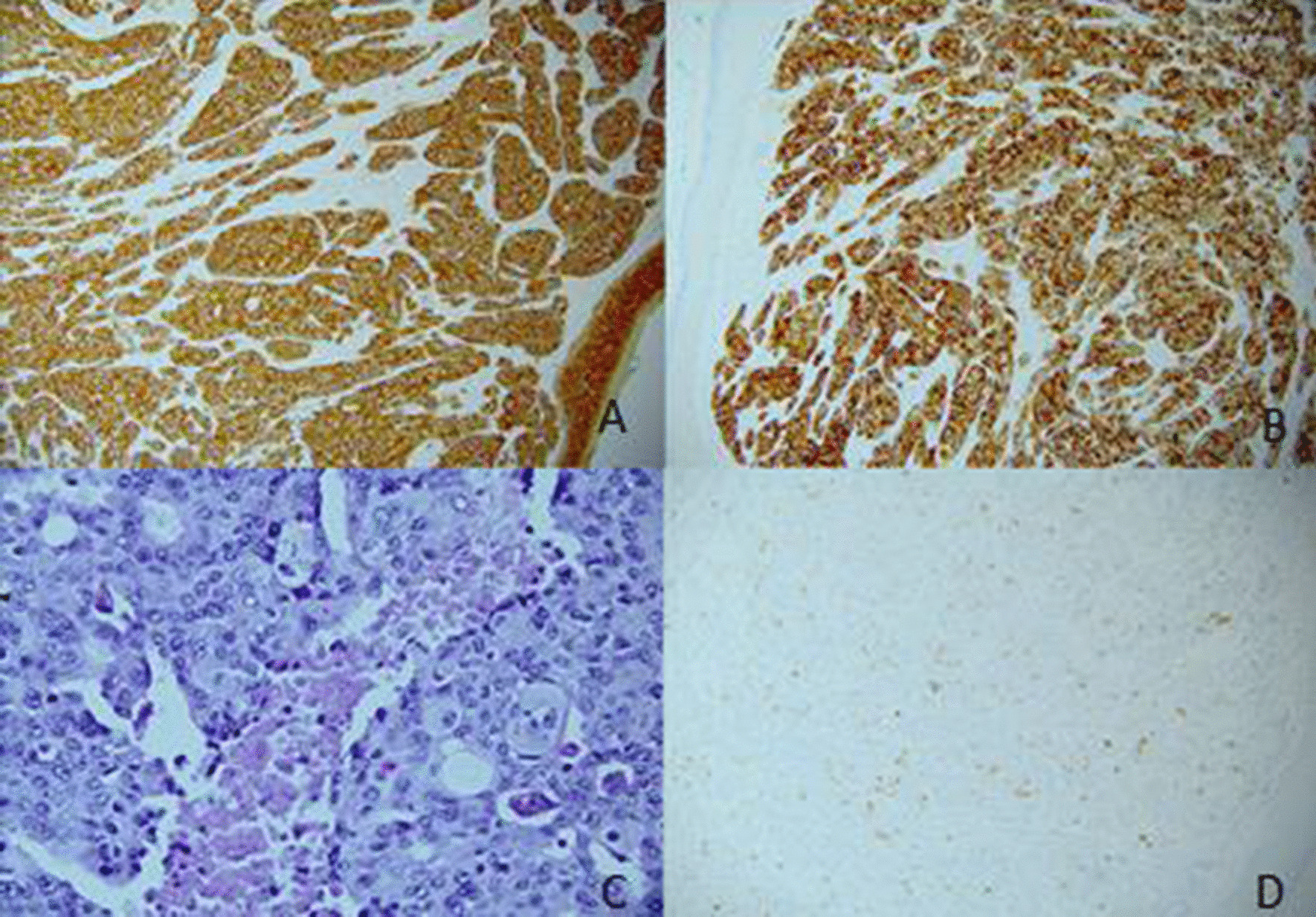
Photomicrograph of breast and scalp lesions shows staining for **a** AE1/AE3, **b** CK 7, **c** focal mucin droplets, and **d** mammaglobin

A 47-year-old Caucasian woman with Eastern Cooperative Oncology Group (ECOG) grade 0 presented with a palpable mass in the left breast associated with an enlarging scalp lesion over 4 months. Biopsy confirmed a diagnosis of metastatic ER/PR positive, HER2-negative breast carcinoma (Fig. 1). Computerized tomography staging demonstrated a multifocal primary lesion fixed to the chest wall, axillary lymphadenopathy, and lung and liver lesions, as well as omental, scalp, and bony involvement. She had no other significant comorbidity. She was started on chemotherapy with nab-paclitaxel, a commonly used agent in the first-line treatment of metastatic breast cancer.

Following three cycles of nab-paclitaxel (260 mg/m^2^ every 21 days each cycle), there was a partial response with shrinkage of tumor in all areas. Her cancer antigen 15-3 declined from 179 to 25 kU/L. She continued with a further three cycles of chemotherapy. Prior to proceeding with the sixth cycle of nab-paclitaxel, she presented with a left-sided lower motor neuron weakness of the face. It was classified as severe as she was unable to close her eyes. There was no evidence of an intracranial lesion or ischemic changes on CT or MRI of the brain. At this point, she was diagnosed with bilateral facial nerve palsy and was administered a trial of oral prednisolone for 5 days without any improvement in her symptoms.

**Fig. 2:**
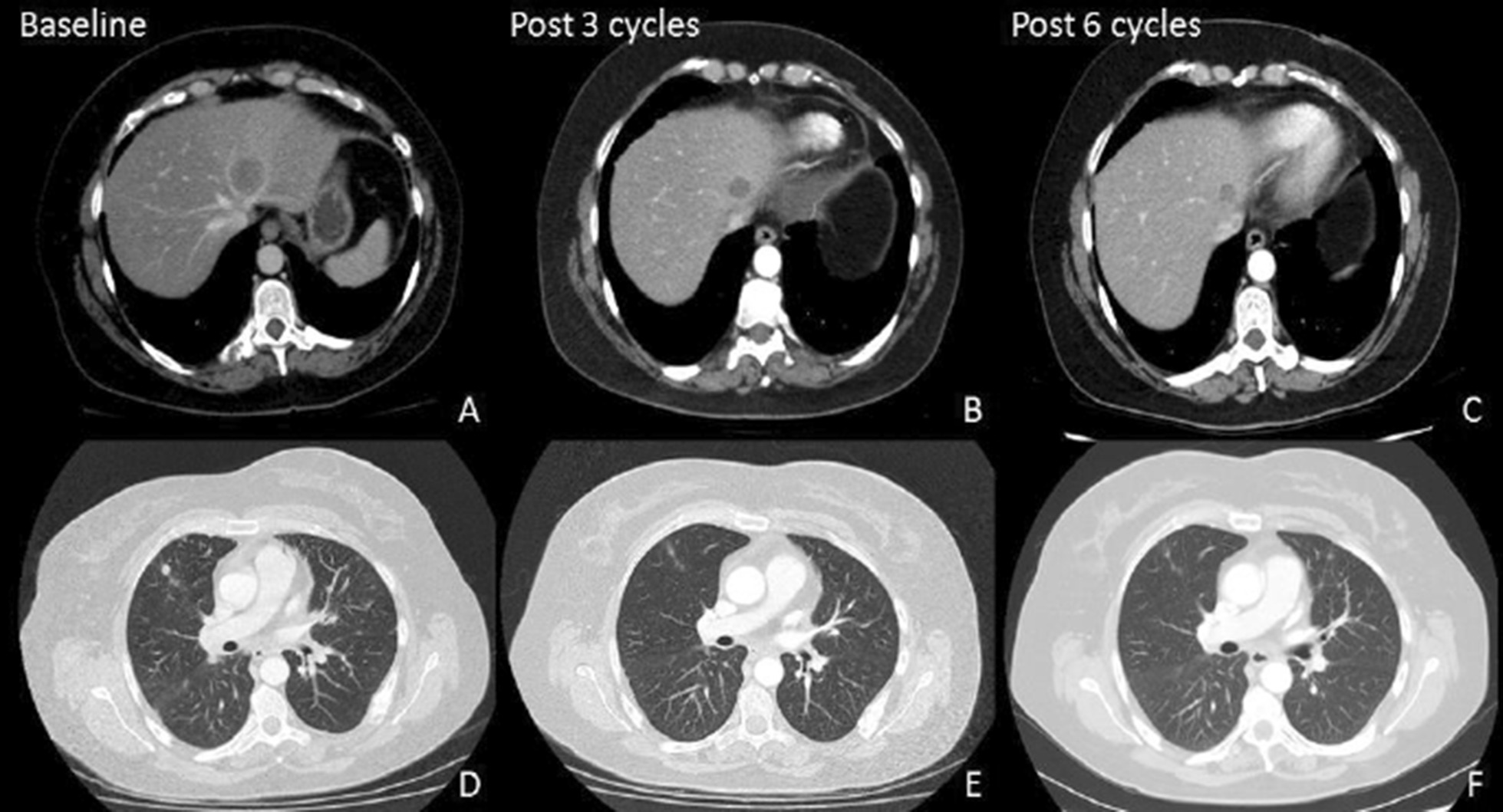
Computerized tomography scans demonstrating reduction in tumor size of the liver (**a**–**c**) and lung (**d**–**f**) metastases after three and six cycles of nab-paclitaxel

One week later, she presented with a lower motor neuron weakness of the contralateral face, giving her bilateral facial nerve palsy. The remainder of the neurological examination did not reveal additional deficits. Subsequent MRI of the brain demonstrated evidence of bilateral facial nerve neuritis involving predominantly the terminal branches. Analysis of the cerebrospinal fluid (CSF) revealed no infective or malignant etiology. Interestingly, the paraneoplastic screening showed the presence of anti-amphiphysin antibodies in both serum and CSF. All other anti-neuronal antibodies, including anti-glutamic acid decarboxylase antibodies, were not detected. A repeat CT scan following the completion of six cycles of chemotherapy demonstrated a partial response according to Response Evaluation Criteria in Solid Tumors (RECIST) 1.1 criteria to the visceral diseases, with a further reduction in cancer antigen 15-3 (Fig. 2).

The patient was started on 1 g IV pulse methylprednisolone for 3 days. This was followed up with intravenous immunoglobulins (IVIG) at a dose of 2 g/kg divided over 5 days. She completed four cycles of IVIG at the 2 g/kg dose, which resulted in a subtle improvement of the frontalis muscle; however, the loss of nasolabial folds and inability to close her eyes persisted. A repeat MRI revealed resolution of facial nerve neuritis. A repeat analysis of CSF showed a high level of anti-amphiphysin antibodies titer of 1:640. Nerve conduction study and electromyography suggested evidence of peripheral nerve reinnervation. She continued with monthly IVIG for the next 6 months. Her chemotherapy was stopped and switched to maintenance hormonal therapy with letrozole 2.5 mg daily to help control her malignant disease. A repeat CT scan 3 months later showed overall stable malignant disease.

## Discussion

PNS is a rare event that affects < 1% of patient with an underlying malignancy [3]. An international panel of neurologists categorize the diagnosis of PNS into two subgroups—“definite” and “possible.” A definite diagnosis can be made when there is a classical or nonclassical neurological syndrome with the presence of onconeural antibodies (that is, amphiphysin antibody), with or without evidence of malignancy. The classical neurological syndromes include encephalomyelitis, limbic encephalitis, subacute cerebellar degeneration, and opsoclonus-myoclonus [7]. A small number of case reports have described the link between amphiphysin antibodies with these classical neurological syndromes in patients with breast cancer and SCLC [8, 9].

Amphiphysin is a nerve-terminal protein that is found in high concentration in the nervous system and is presumed to have a role in synaptic vesicle endocytosis [10, 11]. The anti-amphiphysin autoantibody reacts with 128-kD protein in synaptic vesicles [8]. They are usually seen in both serum and CSF in a subset of patients with stiff-person syndrome (SPS), breast cancer, and SCLC [9]. Around 90% of SPS patients with amphiphysin antibody have been found to have breast cancer during their illness [12].

There has been no published report on the association between the presence of amphiphysin antibodies in breast cancer and bilateral facial nerve palsy. The diagnosis of bilateral facial nerve palsy is itself a rare entity. It is unlikely to be idiopathic in nature and usually reflects an underlying pathology. The potential causes include bilateral acoustic neuroma, Lyme disease, Guillain–Barre syndrome, syphilis, HIV, sarcoidosis, or a tumor, among others [13–15].

Our case highlights an unusual presentation of bilateral facial nerve palsy and a diagnostic dilemma. The potential causes may be either PNS or drug-induced nerve palsy with nab-paclitaxel. The presence of amphiphysin antibody in her blood and CSF despite four cycles of IVIG and a modest response to treatment led us to believe the underlying process to be due to paraneoplastic syndrome. However, the indolent nature and stable state of her breast cancer does not support PNS, as it usually behaves aggressively.

Alternatively, nab-paclitaxel can commonly cause sensory peripheral neuropathy and motor neuropathy [16]. One important risk factor for this is high dosing and frequency of treatment. A case report previously described a case of bilateral facial nerve palsy in metastatic breast cancer following one cycle of high-dose paclitaxel (825 mg/m^2^), which eventually resolved after 23 months [17]. Another case described a case of unilateral facial nerve palsy following nab-paclitaxel (260 mg/m^2^), which improved after 9 months [18]. Though drug-induced facial nerve palsy is a possible cause for our patient’s presentation, her facial nerve palsy has not significantly improved since cessation of nab-paclitaxel. Moreover, it does not explain the presence of amphiphysin antibody in our case.

## Conclusions

Identifying the cause of PNS can sometimes be challenging. Our approach to the case was to stop nab-paclitaxel because of the possibility of its being a direct cause of bilateral facial nerve palsy and switch to endocrine therapy as well as treat the underlying PNS with IVIG and high-dose methyl prednisolone. Currently, there are no guidelines for the management of PNS; however, the principle of treatment suggests treating the underlying tumor with surgery, chemotherapy, or radiotherapy [3]. Other modalities of treatment include immunotherapy with high-dose methyl prednisolone or IVIG.

## Data Availability

Please contact author for data requests

## Abbreviations

CSF: Cerebrospinal fluid
CT: Computerized tomography
HIV: Human immunodeficiency virus
IVIG: Intravenous immunoglobulins
MRI: Magnetic resonance imaging
PNS: Paraneoplastic neurological syndrome
SCLC: Small-cell lung cancer
SPS: Stiff-person syndrome

## References

[1] Vedeler CA, Antoine JC, Giometto B, et al. Paraneoplastic neurological syndromes. In: Gilhus NE, Barnes MP, Brainin M, editors., et al., European Handbook of Neurological Management. 2nd ed. Blackwell Publishing Ltd: Oxford; 2011. p. 447–57.

[2] Honnorat J, Antoine JC. Paraneoplastic neurological syndromes. Orphanet J Rare Dis. 2007;2:22. 10.1186/1750-1172-2-22 PMC1868710 17480225

[3] Kannoth S. Paraneoplastic neurologic syndrome: a practical approach. Ann Indian Acad Neurol. 2012;15(1):6–12. 10.4103/0972-2327.93267 PMC3299076 22412264

[4] De Camilli P, Thomas A, Cofiell R, Folli F, Lichte B, Piccolo G, Meinck H, Austoni M, Fassetta G, Bottazzo G, Bates D, Cartlidge N, Solimena M, Kiliman MW. The synaptic vesicle associated protein amphiphysin is the 128-kD autoan autoantigen of Stiff-Man syndrome with breast cancer. J Exp Med. 1993;178:2219–23. 10.1084/jem.178.6.2219 PMC2191289 8245793

[5] Folli F, Solimena M, Cofiell R, Austoni M, Tallini G, Fassetta G, Bates D, Cartlidge N, Bottazzo GF, Piccolo G, et al. Autoantibodies to a 128-kd synaptic protein in three women with the stiffman syndrome and breast cancer. NEngl J Med. 1993;328:546–51. 10.1056/NEJM199302253280805 8381208

[6] Dropcho EJ. Antiamphiphysin antibodies with small-cell lung carcinoma and paraneoplastic encephalomyelitis. Ann Neurol. 1996;39:659–67. 10.1002/ana.410390516 8619552

[7] Graus F, Delattre JY, Antoine JC, Dalmau J, Giometto B, Grisold W, Honnorat J, Smitt PS, Vedeler Ch, Verschuuren JJ, Vincent A, Voltz R. Recommended diagnostic criteria for paraneoplastic neurological syndromes. J Neurol Neurosurg Psychiatry. 2004;75:1135–40. 10.1136/jnnp.2003.034447 PMC1739186 15258215

[8] Antoine JC, Absi L, Honnorat J, Boulesteix JM, Brouker T, Vial C, Butler M, De Camilli P, Michel D. Antiamphiphysin autoantibodies are associated with various paraneoplastic neurological syndromes and tumors. Arch Neurol. 1999;56(2):172–7. 10.1001/archneur.56.2.172 10025422

[9] Saiz A, Dalmau J, Butler MH, Chen Q, Delattre JY, De Camilli P, Graus F. Anti-amphiphysin I antibodies in patients with paraneoplastic neurological disorders associated with small cell lung carcinoma. J Neurol Neurosurg Psychiatry. 1999;66:214–7. 10.1136/jnnp.66.2.214 PMC1736210 10071102

[10] David C, McPherson PS, Mundigl O, De Camilli P. A role of amphiphysin in synaptic vesicle endocytosis suggested by its binding to dynamin in nerve terminals. Proc Natl Acad Sci U S A. 1996;93:331–5. 10.1073/pnas.93.1.331 PMC40232 8552632

[11] Lichte B, Veh RW, Meyer HE, Kilimann MW. Amphiphysin, a novel protein associated with synaptic vesicles. EMBO J. 1992;11:2521–30. 10.1002/j.1460-2075.1992.tb05317.x PMC556727 1628617

[12] Murinson BB, Guarnaccia JB. Stiff-person syndrome with amphiphysin antibodies. Neurology. 2008;71:1955–8. 10.1212/01.wnl.0000327342.58936.e0 PMC2676978 18971449

[13] Jain V, Deshmukh A, Gollomp S. Bilateral facial paralysis case presentation and discussion of differential diagnosis. J Gen Intern Med. 2006;21(7):C7–10. 10.1111/j.1525-1497.2006.00466.x PMC1924702 16808763

[14] Gevers G, Lemkens P. Bilateral simultaneous facial paralysis—differential diagnosis and treatment options. Acta Otorhinolaryngol Belg. 2003;57:139–46. 12836471

[15] Burakgazi A, Schmidley JW. A rare cause of bilateral facial palsy. Am J Med Case Rep. 2016;4(2):62–4.

[16] Freilich RJ, Balmaceda C, Seidman AD, Rubin M, DeAngelis LM. Motor neuropathy due to docetaxel and paclitaxel. Neurology. 1996;47:115–8. 10.1212/wnl.47.1.115 8710063

[17] Lee RT, Oster MW, Balmaceda C, Hesdorffer CS, Vahdat LT, Papadopoulos KP. Bilateral facial nerve palsy secondary to administration of high-dose paclitaxel. Ann Oncol. 1999;10(10):1245–7. 10.1023/a:1008380800394 10586344

[18] Minatani N, Kosaka Y, Sengoku N, Kikuchi M, Nishimiya H, Waraya M, Enomoto T, Tanino H, Watanabe M. A case of facial nerve palsy induced by nab-paclitaxel. Abstract. Gan To Kagaku Ryoho. 2013;40(12):2375–7. 24394117

